# Gender-Based Association of Coronary Artery Calcification and Framingham Risk Score With Non-alcoholic Fatty Liver Disease and Abdominal Obesity in Taiwanese Adults, a Cross-Sectional Study

**DOI:** 10.3389/fcvm.2022.803967

**Published:** 2022-03-03

**Authors:** Meng-Ting Tsou, Jau-Yuan Chen

**Affiliations:** ^1^Department of Family Medicine, Mackay Memorial Hospital, Taipei City, Taiwan; ^2^Department of Occupation Medicine, Mackay Memorial Hospital, Taipei City, Taiwan; ^3^Department of Mackay Junior College of Medicine, Nursing, and Management, New Taipei City, Taiwan; ^4^Department of Family Medicine, Chang-Gung Memorial Hospital, Linkou Branch, Linkou, Taiwan; ^5^Department of Medicine, College of Medicine, Chang Gung University, Taoyuan, Taiwan

**Keywords:** coronary artery calcification, non-alcoholic fatty liver disease, abdominal obesity, Framingham risk score, Taiwan

## Abstract

**Background:**

It is not certain whether non-alcoholic fatty liver disease (NAFLD) or abdominal obesity (AO) has stronger associations with atherosclerosis and coronary artery disease (CAD) risk across different genders. The purpose of this study was to determine the gender-based association of NAFLD and AO with subclinical atherosclerosis represented by coronary artery calcification (CAC) and CAD risk by Framingham risk score (FRS).

**Methods:**

A total of 1,655 participants in a health-screening program (mean age: 49.44 years; males: 70.33%) were enrolled for analysis. Fatty liver and coronary artery calcium score (CACS) were measured via ultrasonography (US) and multi-detector computed tomography (MDCT). The presence of CAC was defined as having a CACS > 0, intermediate to high CAD risk was defined as FRS ≥ 10%, while the presence of AO was defined as having a waist circumference (WC) of ≥90 cm for men and ≥80 cm for women. Participants were categorized into four groups depending on the presence or absence of NAFLD and/or AO.

**Results:**

The percentage of subjects with CACS > 0 was highest in the AO-only group (overall: 42.6%; men: 48.4%; women: 35.8%); and FRS ≥ 10% was highest in the group with both abnormalities (overall: 50.3%%; men: 57.3%; women: 32.4%). After adjustment factors, the odds ratio (OR) for CAC and FRS was the highest in the group with both abnormalities [men: 1.61 (1.13–2.30) for CACS > 0 and 5.86 (3.37–10.20) for FRS ≥ 10%; women: 2.17 (1.13–4.16) for CACS > 0 and 6.31 (2.08–19.10) for FRS ≥ 10%]. In men, the OR of NAFLD was higher than that of AO [1.37 (1.03–1.83) vs. 1.35 (1.02–1.79) for CACS > 0, 3.26 (2.13–4.98) vs. 2.97 (1.91–4.62) for FRS ≥ 10%]. However, women with AO consistently showed increased OR for CACS > 0 [1.87 (1.11–3.16)] and FRS ≥ 10% [4.77 (2.01–11.34)].

**Conclusion:**

The degree of association of NAFLD and AO with CAC and FRS depends on the gender. NAFLD is more closely associated with CACS > 0 and FRS ≥ 10% in men and AO in women, respectively. NAFLD and AO could be considered independent determinants of CAC and FRS by gender.

## Introduction

Coronary artery disease (CAD) caused by atherosclerosis is the leading cause of morbidity and mortality worldwide, thus becoming one of the most serious global health issues (1). In asymptomatic individuals, early detection of subclinical atherosclerosis is necessary to prevent or delay its progression to overt CAD. Coronary artery calcium score (CACS), a useful marker of subclinical atherosclerosis detected by multi-detector computed tomography (MDCT), is frequently used to predict and prevent overt CAD (2). The close correlations of CACS with cardiovascular events or obesity have already been noted in a previous study (3). The Framingham risk score (FRS) provides an estimate of CAD risk for the general population (4–6). As mentioned in previous studies, both FRS and CACS were independently associated with subclinical atherosclerosis in asymptomatic subjects with low to intermediate cardiovascular risk (5, 6).

Obesity is regarded as an independent risk factor for atherosclerosis and CAD (7, 8), and is closely associated with other CAD risk factors such as hypertension (HTN), diabetes mellitus (DM), and dyslipidemia (9, 10). Previous studies have focused on abdominal and visceral obesity (localized distribution of body fat rather than total body fat), which have since been considered major risk factors for CAD (11–14). From the recent study on severe acute respiratory coronavirus syndrome 2 (SARS-CoV-2), it was found that obesity (specifically visceral obesity) and characteristics of impaired metabolic health such as DM, HTN and subclinical inflammation, also emerged as important determinants of severe coronavirus disease 2019 (COVID-19) (15). Waist circumference (WC) and hip circumference (HC) are two of the easy ways to measure abdominal obesity (AO) (16, 17). Actually, WC allows visceral adiposity to be differentiated from total obesity, considers fat distribution, correlates well with abdominal imaging, and is more predictive of coronary artery calcification (CAC) than other anthropometric indicators (16, 18, 19).

Non-alcoholic fatty liver disease (NAFLD) has become a common health problem with a prevalence of about 20–30% in Western population (20); among extensive population-based surveys in Asia, the prevalence rates of NAFLD were between 2.04 and 52% (21, 22). Taiwan has a prevalence of 11.5–52% (23). NAFLD is a manifestation of metabolic syndrome (MetS), obesity, and insulin resistance (IR) in the liver (24, 25). Previous studies have shown that NAFLD could be considered a cardiovascular risk factor by advancing the progress of subclinical atherosclerosis or cardiovascular disease (26–28). However, it is unclear whether there is gender-based differences in the association of NAFLD or WC with atherosclerosis and CAD risk. Therefore, risk assessment of NAFLD and WC by gender is important to apply different therapeutic approaches to reduce atherosclerosis and reduce the risk of CAD.

A recent study suggests that NAFLD is more closely related with CAC than AO assessed by waist-to-hip ratio (WHR) (29). In addition, whether a cross-sectional study or a cohort study, they reported a predicted higher 10-year CAD risk as determined by the FRS in patients with NAFLD (30, 31). The purpose of this study is to determine gender differences in the association of NAFLD and AO and the risk of subclinical atherosclerosis and CAD in an apparently healthy Taiwanese population. For this purpose, we evaluated the risk of subclinical atherosclerosis and CAD in subjects divided into four groups according to the presence/absence of NAFLD diagnosed by US and the presence/absence of AO measured via WC status in men and women. We analyzed CACS and FRS in these four groups to determine the association of NAFLD and AO with risk of subclinical atherosclerosis and CAD in men and women.

## Method

### Study Design and Study Population

**Figure 1:**
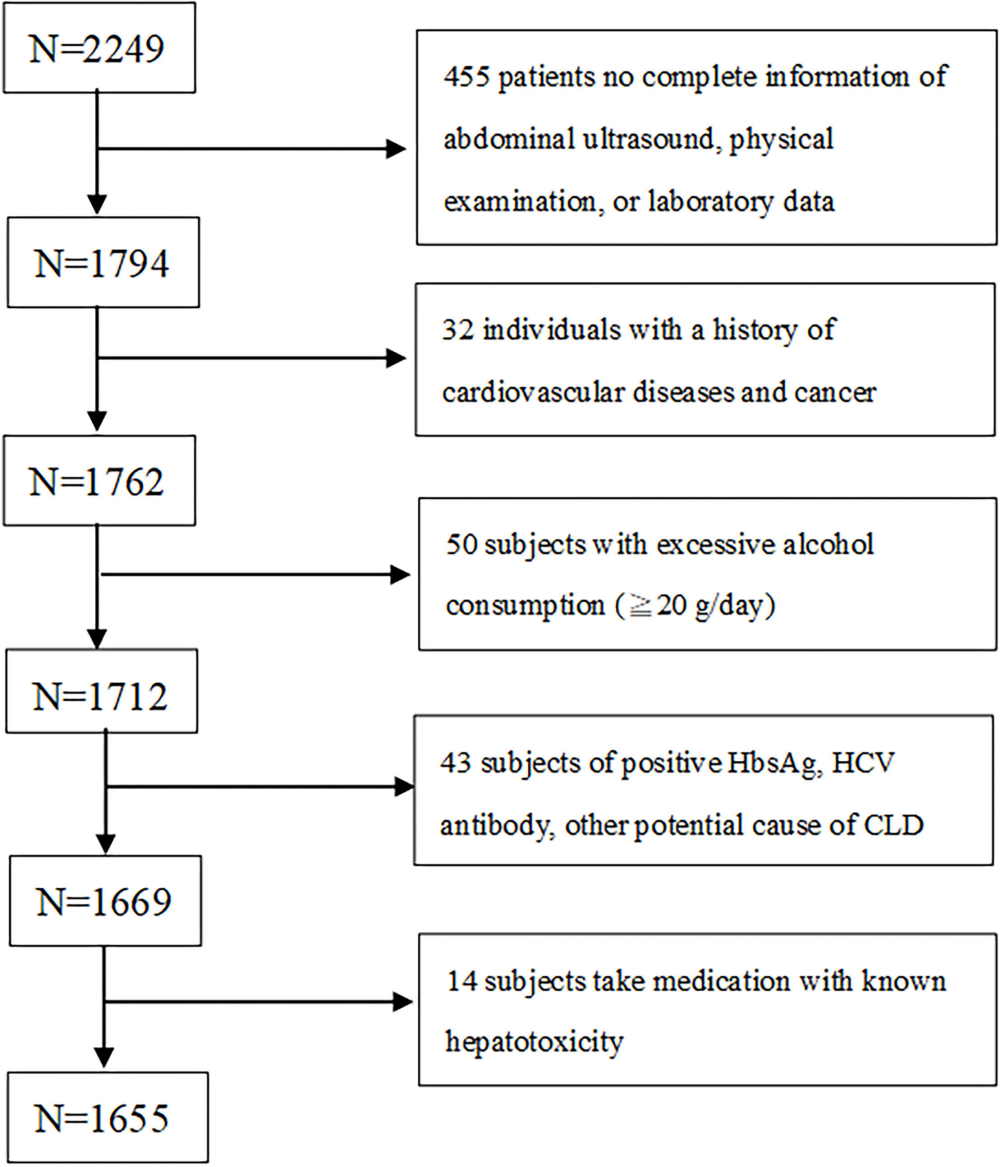
Flow chart of enrolled participants meet the requirements.

In this cross-sectional study, which was performed retrospectively from 2005 to 2009, 2,249 adult subjects participated. They were screened for cardiovascular health at a territory medical center in Taipei, where they were subjected to MDCT to screen their coronary calcium levels. Subjects who had full details about abdominal ultrasound (US), height, weight, body mass index (BMI), waist circumference (WC), information on the metabolic component were selected for the study. All the subjects had a self-reported history of acute ischemic heart disease, angina pectoris or any congenital heart disease. Subjects who didn't fulfill these two selection criteria were excluded. Further subjects who consumed alcohol ≥20 g/day, history of viral hepatitis or chronic hepatic disease, consumption of hepatoxicity medicine (like statins, estrogen, tamoxifen, diltiazem, and valproate) were all excluded from the study (Figure 1). After applying the selection criteria 594 subjects were excluded and finally, 1,655 (age: 49.5 ± 9.8 years; 70.3 % male) were selected for the study.

A standardized questionnaire was used to screen the baseline characteristics, medical history and other intricate information open through physical examination. We considered a systolic blood pressure (SBP) of more than 140 mmHg, and/or diastolic blood pressure (DBP) of more than 90 mmHg as hypertensive. Subjects who were already diagnosed as hypertensive and under medication were also considered. Fasting Glucose (FG) level of 126 mg/dL or consumption of oral hypoglycemic agent/insulin were considered diabetic in the study. Subjects who had total cholesterol (TC) levels more than 200 mg/dL or triglycerides (TG) more than 150 mg/dL or under medication for dyslipidemia were considered dyslipidemia in this study. A part of this research had been published already in 2012 (32).

### Anthropometric and Laboratory Measurement

At the baseline, a complete anthropometric analysis was carried out where height, weight, BMI and WC were recorded. WC was assessed following normal expiration from the midpoint between the 12th rib and the iliac crest. A standard sphygmomanometer was used to define resting blood pressure. All the anthropometric values for the study were collected at a laboratory center by well-trained nurses who were blinded to the study participants.

Acquisition and analysis - Hitachi 7170 Automatic Analyzer (Hitachi Corp., Hitachinaka Ibaraki, Japan) was used for measuring FG (hexokinase method) following 12 h of fasting before morning 10 AM. Along with this complete lipid profiles [including TC, TGL, low and high-density lipoprotein (LDL, HDL); homogenous enzymatic colorimetric assay], aspartate aminotransferase (AST), alanine aminotransferase (ALT), hepatitis B surface antigen, and an antibody to hepatitis C virus were also assessed using standard laboratory procedures.

### Diagnosis of NAFLD and Abdominal Obesity

Fatty liver was diagnosed using abdominal US (Acuson, Sequoia 512, Siemens, Mountain View, CA) performed by gastroenterologists richly experienced who were blinded to the laboratory and clinical details of the subjects at the time of the procedure. If different results were found, a third doctor was asked to perform the test while blinded to test results and patient information. Asia-Pacific Working Party on NAFLD and Chinese Association for the Study of Liver Disease (CASLD) guidelines were adopted. The presence of at least two of the following factors was considered fatty liver; diffusely enlarged liver near field ultrasound echo (“bright liver”); liver echo greater than kidney; vascular blurring and the gradual attenuation of far-field ultrasound echo (33, 34).

Subjects were rendered AO if their waist circumference ≥ 90 cm among men and 80 cm among women in accordance to the classification of the National Cholesterol Education Panel of the National Treatment Program for Adults III (NCEP-ATP III) Taiwanese population for AO (35, 36).

### CAC Measurements

**Figure 2:**
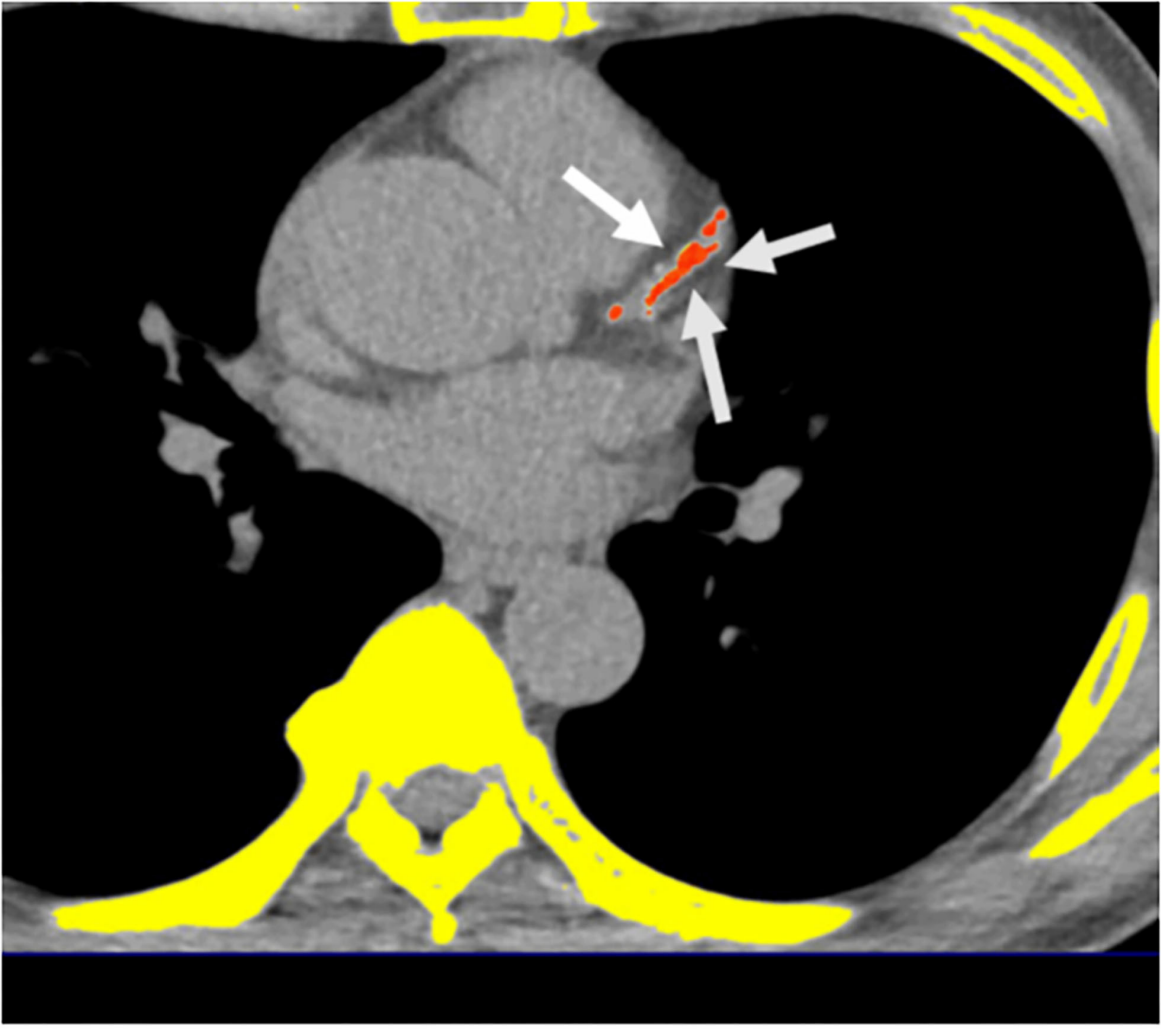
Multidetector computed tomography (MDCT) demonstrated the quantification of CACS. Semi-automatic quantification of CACS burden using Agatston scoring. *Orange color regions indicate visceral fat tissue. White arrows indicate coronary calcification lesions.

A 16-slice MDCT scanner (Sensation 16; Siemens Medical Solutions, Forchheim, Germany) with 16- ×0.75-mm collimation, rotation time of 420 ms, and tube voltage of 120 kV was used to measure the calcification of all coronary arteries using a dedicated offline workstation (Aquarius 3D Workstation; TeraRecon, San Mateo, CA, USA). A site with a density >130 HU and lasting at least six pixels were defined as a “coronary calcified lesion.” Distinct atherosclerosis that was clinically important was defined by the presence of CAC; the burden of atherosclerosis that was defined by CAC score (CACS) which was semi-quantitatively measured using the Agatston score by multiplying every lesion (area) by a weighted CT attenuation score of the lesion (Figure 2) (32).

### FRS Score Calculation and Risk Category

The FRS analysis after considering the subjects age, gender, TC, HDL, SBP, treatment for hypertension and cigarette smoking (36) categorized them as “low” (<10% 10-year risk), “intermediate” (10–20% risk) or “high” risk (≧ 20% risk).

### Ethics

Ethical clearance for the study (done per the Helsinki Declaration of 1975) was provided by the Human Research Ethics Committee of Mackay Memorial Hospital (project research number 18MMHIS137, 15 Oct 2018). The subjects were de-identified for data analysis and didn't give any informed consent as this was a retrospective study.

### Statistical Analysis

The baseline values were presented as mean ± SD for continuous data and percentile value for categorical data. If the normality assumption was violated based Shapiro-Wilk test, we use the non-parametric method instead. Non-parametric comparisons of the medians between the groups were performed using Kruskal–Wallis H test. One-way analysis of variance was used to analyze parametric data and chi-square or Fisher's exact test was used for non-parametric data analysis. Because this study aimed to further realize the difference between the two groups, separately, the data was mainly analyzed with *post-hoc* analysis with Bonferroni correction.

As CACS values were extremely skewed, logarithmized CACS + 1 was used for the comparison between the groups. CACS and FRS were dichotomized as the presence of CACS > 0 vs. absence of CACS = 0 and FRS ≥ 10 vs. FRS <10% for binary logistic regression analysis. Multivariate logistic regression analyses were performed to analyze the relationship among groups with CACS > 0 and FRS ≥ 10% while controlling for potential confounding variables included in the model. Covariates in the multivariable model, chosen for clinical importance as well as statistical significance included age, BMI, HTN, DM, hyperlipidemia, cigarette smoking, alcohol drinking, and exercise. SPSS (IBM Corp., Armonk, NY, USA) was used to compute the statistic for the study. Odds ratios (ORs) was obtained and a significance level was fixed at *p* < 0.05 at 95% confidence intervals (CIs).

## Results

### The General Characteristics of Study Participants by Gender

A total of 1,655 participants, including 1,164 men (70.3%, mean age 48.67 ± 9.61 years) and 491 women (29.7%, 51.26 ± 9.88 years) were classified into four groups according to the presence/absence of NAFLD and AO (Table 1) as follows: (1) subjects without either abnormality (men, *n* = 347, 29.8%; women, *n* = 198, 40.4%); (2) subjects with AO only (men, *n* = 62, 5.3%; women, *n* = 53, 10.8%); (3) subjects with NAFLD only (men, *n* = 404, 24.7%; women, *n* = 101, 20.6%); and (4) subjects with both abnormalities (men, 351, 30.2%; women, *n* = 139, 30.2%).

**Table 1:** The general characteristics of study participants by gender.

**Variables**	**Total population**	**NAFLD (−)**	**NAFLD (−)**	**NAFLD (+)**	**NAFLD (+)**	***p*-value**	**Shapiro-Wilk**
		**AO (−)**	**AO (+)**	**AO (−)**	**AO (+)**		**Normality Test**
**Men**	**(*****n*** **=** **1,164, 70.3%)**	**(*****n*** **=** **347, 29.8%)**	**(*****n*** **=** **62, 5.3%)**	**(*****n*** **=** **404, 24.7%)**	**(*****n*** **=** **351, 30.2%)**		
Age (year)	48 [42, 54]	48 [41, 54]	50 [43.75, 59]^a^	47 [41, 53]^b^	50 [43, 56]^c^	0.001	<0.001
BMI (kg/m^2^)	24.71 [22.9, 27.0]	22.6 [21.3, 24.2]	27.25 [25.27, 28.92]^a^	24 [22.90, 25.53]^a^, ^b^	28.03 [26.42, 31.10]^a^, ^b^, ^c^	<0.001	<0.001
WC (cm)	87 [81, 92]	81 [76, 85]	93 [91, 96.25]^a^	84 [80, 87]^a^, ^b^	95 [92, 100]^a^, ^b^, ^c^	<0.001	<0.001
SBP (mmHg)	120 [110, 132]	120 [110, 130]	129 [113, 138.5]^a^	120 [110, 130]^b^	130 [118, 138]^a^, ^c^	<0.001	<0.001
DBP (mmHg)	78 [70, 84]	74 [70, 80]	80 [70, 88.5]^a^	76 [70, 82]^b^	80 [76, 88]^a^, ^c^	<0.001	<0.001
FPG (mg/dL)	98 [92, 105]	94 [90, 101]	99 [92, 106]	97 [92, 102]	102 [96, 116]^a^, ^b^, ^c^	<0.001	<0.001
TC (mg/dL)	201 [178, 225]	197 [175, 224]	192.5 [166.5, 212.0]	203 [180, 225]	207 [181, 228]^a^	0.01	<0.001
TG (mg/dL)	126 [89.25, 184.75]	96 [72, 137]	122.5 [93.75, 183.25]	125.5 [91.25, 174.00]^a^	162 [114, 243]^a^, ^c^	<0.001	<0.001
HDL-C (mg/dL)	47 [40, 55]	52 [45, 62]	44 [38.5, 51.0]^a^	48 [41, 56]^a^	43 [37, 49]^a^, ^c^	<0.001	<0.001
LDL-C (mg/dL)	133 [112, 154]	128 106.75, 151	130 [103, 147]	135 [115, 157]	136 [115.75, 155.00]^a^, ^b^, ^c^	0.01	0.23
AST (IU/L)	23 [19, 29]	21 [18, 25]	22 [19.75, 27.25]	23 [19, 28]	26 [22, 35]^a^, ^c^	<0.001	<0.001
ALT (IU/L)	27 [20, 40]	21 [17, 27]	26 [20.5, 38.25]	28 [21, 40]^a^	36 [27, 53]^a^, ^b^, ^c^	<0.001	<0.001
Smoking, *n* (%)	220 (18.9)	53 (15.3)	12 (19.4)	78 (19.3)	77 (21.9)	0.16	
Alcohol drinking, *n* (%)	90 (7.7)	20 (5.8)	5 (8.1)	28 (6.9)	37 (10.5)	0.11	
Exercise, *n* (%)	209 (18.0)	62 (17.9)	10 (16.1)	76 (18.8)	61 (17.4)	0.94	
HTN, *n* (%)	231 (19.8)	50 (14.4)	21 (33.9)^a^	56 (13.9)	104 (29.6)^a^, ^c^	<0.001	
DM, *n* (%)	81 (7.0)	22 (6.3)	5 (8.1)	16 (4.0)^a^	38 (10.8)^c^	0.003	
Hyperlipidemia, *n* (%)	69 (5.9)	15 (4.3)	3 (4.8)	19 (4.7)	32 (9.1)	0.03	
FRS ≥ 10% (intermediate to high CVD risk), *n* (%)	480 (41.2)	106 (30.5)	33 (53.2)	140 (34.7)	201 (57.3)	<0.001	
**Women**	**(*****n*** **=** **491, 29.7%)**	**(*****n*** **=** **198, 40.4%)**	**(*****n*** **=** **53, 10.8%)**	**(*****n*** **=** **101, 20.6%)**	**(*****n*** **=** **139, 30.2%)**		
Age (year)	52 [45, 57]	48.5 [41, 55]	58 [50.5, 64.0]^a^	49 [43, 56]^b^	54 [49, 60]^a^, ^c^	<0.001	0.045
BMI (kg/m^2^)	22.90 [20.92, 25.43]	20.8 [19.6, 22.2]	24.41 [23.0, 26.2]^a^	22.87 [21.5, 24.0]^a^, ^b^	26.48 [24.8, 28.9]^a^, ^b^, ^c^	<0.001	<0.001
WC (cm)	77 [72, 84]	71 [68, 75]	84 [81, 89]^a^	75 [72, 78]^a^, ^b^	87 [82, 93]^a^, ^b^, ^c^	<0.001	<0.001
SBP (mmHg)	118 [108, 130]	110 [102, 122]	124 [110, 132]^a^	116 [108, 130]	122 [112, 138]^a^, ^c^	<0.001	<0.001
DBP (mmHg)	72 [68, 80]	70 [64, 75]	74 [70, 82]^a^	70 [67, 80]^a^	80 [70, 82]^a^, ^c^	<0.001	<0.001
FPG (mg/dL)	94 [89, 101]	91 [87, 96]	96 [90, 100]	93 [88.5, 98.5]	101 [94, 112]^a^, ^b^, ^c^	<0.001	<0.001
TC (mg/dL)	203 [176, 228]	194.5 [169.75, 216.00]	204 [176.0, 230.5]	204 [174.5, 229]^a^	212 [186, 246]^a^, ^b^, ^c^	<0.001	<0.001
TG (mg/dL)	94 [67, 137]	75.5 [61, 101]	89 [73, 123]	98 [76, 138.5]^a^	142 [102, 190]^a^, ^b^, ^c^	<0.001	<0.001
HDL-C (mg/dL)	60 [51, 69]	66 [56, 76]	61 [53, 68]^a^	57 [49, 65.5]^a^	55 [45, 62]^a^, ^b^	<0.001	<0.001
LDL-C (mg/dL)	124 [102, 150]	114 [95.5, 137.5]	124 [104.5, 149.5]	128 [103.5, 150.5]^a^	136 [111, 167]^a^	<0.001	<0.001
AST (IU/L)	21 [17, 25]	19 [17, 23]	21 [17, 25]	21 [18, 25]	22 [19, 28]^a^	<0.001	<0.001
ALT (IU/L)	19 [14, 25]	16 [13, 21]	18 [14, 25]	20 [14, 24]	24 [17, 34]^a^, ^b^, ^c^	<0.001	<0.001
Smoking, *n* (%)	10 (2.0)	3 (1.5)	0 (0.0)	3 (3.0)	4 (2.9)	0.57	
Alcohol drinking, *n* (%)	13 (2.6)	4 (2.0)	0 (0.0)	3 (3.0)	6 (4.3%)	0.41	
Exercise, *n* (%)	68 (13.8)	30 (15.2)	12 (22.6)	15 (14.9)	11 (7.9)^a^	0.048	
HTN, *n* (%)	78 (15.9)	13 (6.6)	15 (28.3)^a^	14 (13.9)	36 (25.9)^a^	<0.001	
DM, *n* (%)	29 (5.9)	4 (2.0)	4 (7.5)	6 (5.9)	15 (10.8)^a^	0.01	
Hyperlipidemia, *n* (%)	31 (6.3%)	4 (2.0)	5 (9.4)	7 (6.9)	15 (10.8)^a^	0.01	
FRS ≥ 10% (intermediate to high CVD risk), *n* (%)	80 (16.3)	8 (4.0)	13 (24.5)	14 (13.9)	45 (32.4)	<0.001	

*NAFLD, non-alcoholic fatty liver disease; AO, abdominal obesity; BMI, body mass index; WC, waist circumference; SBP, systolic blood pressure; DBP, diastolic blood pressure; FPG, fasting plasma glucose; TC, total cholesterol; TG, Triglyceride; HDL-C, high density lipoprotein cholesterol; LDL-C, low density lipoprotein cholesterol; AST, Aspartate Aminotransferase; ALT, Alanine Aminotransferase; HTN, hypertension; DM, diabetes mellitus; CACS, coronary artery calcium score; FRS, Framingham risk score*.

*Clinical characteristics are expressed as median [Q1, Q3] for continuous variables and n (%) for categorical variables. P-value was derived from Kruskal-Wallis one-way analysis of variance (one-way ANOVA) for continuous variables and categorical variables were expressed as percentages and compared with the x^2^ test or Fisher's exact test*.

a *p < 0.05 vs. NAFLD (−) AO (−)*.

b *p < 0.05 vs. NAFLD (−) AO (+)*.

c *p < 0.05 vs. NAFLD (+) AO (−) in the Bonferroni post-hoc comparisons*.

### Comparison of Factors Between Groups According to NAFLD and AO Status

Comparison of factors between groups revealed that the AO-only group was the oldest, while the worst metabolic factors were found in the group with both abnormalities (Table 1). By gender, metabolic factors, especially lipid profile, were worse in the NAFLD-only group than in the AO-only group. The mean WC for the entire population was 84.45 ± 10.07 cm; AO subjects were generally more obese with higher mean BMI compared to those with NAFLD in both genders (Table 1). The group with both abnormalities had the highest proportion of subjects with abnormal liver function tests, DM, HTN, and dyslipidemia. The proportion of subjects with high ALT, DM, HTN, and dyslipidemia was higher in the AO-only group than in the NAFLD-only group. Furthermore, subjects with NAFLD tended to exercise less than subjects without NAFLD among the women (Table 1).

### Comparison of FRS in Four Groups by NAFLD and AO Status

Among the total population, 33.8 % had intermediate to high risk (FRS ≥ 10%) (men: 41.2%, women: 16.3%). The proportion of subjects with intermediate to high risk was highest in the group with both abnormalities and lowest in the group without either abnormality (overall: 50.3 vs. 21.0%; men: 57.3 vs. 30.5%; women: 32.4 vs. 4.0%) (Table 1).

**Figure 3:**
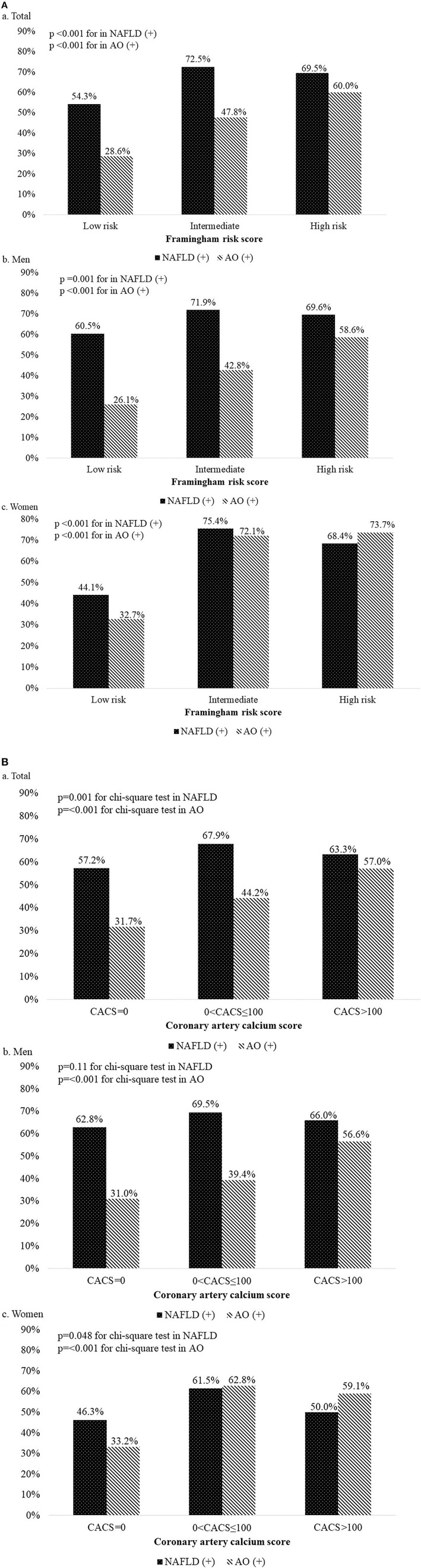
**(A)** Prevalence of non-alcoholic fatty liver disease and abdominal obesity according to the Framingham risk score grade. a, Total participant; b, Men; c, Women. **(B)** Prevalence of non-alcoholic fatty liver disease and abdominal obesity according to the coronary artery calcium score grade. a, Total participant; b, Men; c, Women.

Among all the participants, 995 (60.1%) had NAFLD, while 605 (36.6%) had AO. The prevalence of AO increased from 28.6% (*n* = 311) in subjects with low risk to 47.8% (*n* = 172) in subjects with intermediate risk and 60.0% (*n* = 120) in subjects with high risk (Figure 3A). The same trend was found in men (*n* = 177, 26.1%; *n* = 128, 42.8%; and *n* = 106, 58.6%, respectively) and women (*n* = 134, 32.7%; *n* = 44, 72.1%; and *n* = 14, 73.7%, respectively). Subjects with intermediate risk had the highest prevalence of NAFLD compared with the other groups; this was statistically significant in the overall population and in different gender (total, men, women: 172, 72.5%; 215, 71.9%; 44, 75.4%) (Figure 3A).

### Comparison of CACS in Four Groups by NAFLD and AO Status

Among the total population, 30.7% had CACS > 0 (men: 35.1%, women: 20.4%), and the mean ln(CACS + 1) was 1.02 (men: 1.16; women: 0.67) (Table 2). When comparing the mean, age-adjusted, or age/sex-adjusted ln(CACS + 1) across groups, the values were the highest in the AO-only group and the lowest in the group without either abnormality (Table 2). The proportion of subjects with CAC was highest in the AO-only group and lowest in the group without either abnormality (overall: 42.6 vs. 22.0%; men: 48.4 vs. 28.2%; women: 35.8 vs. 11.1%).

**Table 2:** Comparison of coronary artery calcium score among the four groups divided by non-alcoholic fatty liver disease and abdominal obesity status.

**Variables**	**Total population**	**NAFLD (−)**	**NAFLD (−)**	**NAFLD (+)**	**NAFLD (+)**	***p*-value**
		**AO (−)**	**AO (+)**	**AO (−)**	**AO (+)**	
**Total**	**(*n* = 1,655)**	**(*n* = 545)**	**(*n* = 115)**	**(*n* = 505)**	**(*n* = 490)**	
Mean Ln (CACS+1)	1.02 ± 1.84	0.70 ± 1.58	1.59 ± 2.21^a^	0.87 ± 1.65^b^	1.39 ± 2.08^a^, ^c^	<0.001
Age-adjusted mean Ln (CACS+1)	1.02 ± 0.73	0.93 ± 0.73	1.37 ± 0.82^a^	0.91 ± 0.65^b^	1.15 ± 0.74^a^, ^b^, ^c^	<0.001
Age and sex adjusted mean Ln (CACS+1)	1.02 ± 0.79	0.87 ± 0.83	1.28 ± 0.85^a^	0.97 ± 0.72^b^	1.16 ± 0.78^a^, ^c^	<0.001
CACS > 0, *n* (%)	508 (30.7%)	120 (22.0%)	49 (42.6%)^a^	147 (29.1%)^b^	19 (39.2%)^a^, ^c^	<0.001
**Men**	**(*****n*** **=** **1,164)**	**(*****n*** **=** **347)**	**(*****n*** **=** **62)**	**(*****n*** **=** **404)**	**(*****n*** **=** **351)**	
Mean Ln (CACS+1)	1.16 ± 1.94	0.91 ± 1.77	1.79 ± 2.32^a^	0.96 ± 1.71^b^	1.54 ± 2.18^a^, ^c^	<0.001
Age-adjusted mean Ln (CACS+1)	0.96 ± 0.72	0.92 ± 0.74	1.20 ± 0.80^a^	0.88 ± 0.64^b^	1.05 ± 0.74^c^	<0.001
Age and sex adjusted mean Ln (CACS+1)	1.16 ± 0.76	1.12 ± 0.78	1.42 ± 0.84^a^	1.08 ± 0.68^b^	1.26 ± 0.78^c^	<0.001
CACS > 0, *n* (%)	408 (35.1%)	98 (28.2%)	30 (48.4%)^a^	131 (32.4%)^b^	149 (42.5%)^a^, ^c^	<0.001
**Women**	**(*****n*** **=** **491)**	**(*****n*** **=** **198)**	**(*****n*** **=** **53)**	**(*****n*** **=** **101)**	**(*****n*** **=** **139)**	
Mean Ln (CACS+1)	0.67 ± 1.52	0.34 ± 1.10	1.35 ± 2.08^a^	0.51 ± 1.35^b^	1.00 ± 1.74^a^	<0.001
Age-adjusted mean Ln (CACS+1)	1.15 ± 0.74	0.94 ± 0.70	1.58 ± 0.81^a^	1.04 ± 0.68^b^	1.38 ± 0.68^a^, ^c^	<0.001
Age and sex adjusted mean Ln (CACS+1)	0.67 ± 0.78	0.45 ± 0.74	1.12 ± 0.85^a^	0.55 ± 0.71^b^	0.91 ± 0.72^a^, ^c^	<0.001
CACS >0, n (%)	100 (20.4%)	22 (11.1%)	19 (35.8%)^a^	16 (15.8%)^b^	43 (30.9%)^a^, ^c^	<0.001

*Clinical characteristics are expressed as mean ± SD for continuous variables and n (%) for categorical variables. P-value was derived from one-way analysis of variance (one-way ANOVA) for continuous variables and categorical variables were expressed as percentages and compared with the x^2^ test*.

a *p < 0.05 vs. NAFLD (−) AO (−)*.

b *p < 0.05 vs. NAFLD (−) AO (+)*.

c *p < 0.05 vs. NAFLD (+) AO (−) in the Bonferroni post-hoc comparisons. Abbreviations as list in Table 1*.

Among all the participants, 995 (60.1%) had NAFLD, while 605 (36.6%) had AO. The prevalence of AO increased from 31.7% (*n* = 364) in subjects with CACS = 0 to 44.2% (*n* = 168) in subjects with 0 < CACS ≤ 100 and 57% (*n* = 73) in subjects with CACS > 100 (Figure 3B). The same trend was found in men (*n* = 234, 31%; *n* = 119, 39.4%; and *n* = 60, 56.6%, respectively). However, in women, the highest prevalence of AO was noted in subjects with 0 < CACS ≤ 100 (49, 62.8%). Subjects with 0 < CACS ≤ 100 had the highest prevalence of NAFLD compared with the other groups; this was statistically significant in the overall population and in women, but not in men (total, women, men: 258, 67.9%; 210, 69.5%; 48, 61.5%) (Figure 3B).

### Risk for Intermediate to High CAD Risk (FRS ≥ 10%) in Subjects With Either NAFLD or AO

In the univariate analyses, the OR for FRS ≥ 10% was much higher in the NAFLD group than in the AO group in men, but not in total and women. In the adjusted model, the OR of FRS ≥ 10% in subjects with NAFLD increased compared to subjects without NAFLD in the overall population and in men (Table 3A). The OR for FRS ≥ 10% was higher in subjects with AO compared with those without AO in women (Table 3A). In the multivariable analyses, after adjustment for age, DM, HTN, hyperlipidemia history, cigarette smoking, alcohol drinking, and exercise, the OR of the NAFLD and AO groups was attenuated, whereas the NAFLD group showed a relatively increased risk for FRS ≥ 10% compared to that without NAFLD, and the OR was higher than that in men with AO as well [3.26; 95% confidence interval (CI) 2.13–4.98 vs. 2.97; 95% CI 1.91–4.62] (Table 3A). However, women with AO and NAFLD consistently had an significant statistical OR for FRS ≥ 10%, but OR was higher in the AO group than in the NAFLD group (Table 3A). In the supplementary Table 1A, after adjusting BMI variable, the results still had statistical difference. The variance inflation factor (VIF) for each independent variable by checking multi-collinearity did not reach collinearity.

**Table 3A:** Odds ratio for Framingham risk score ≥10% in subjects with either non-alcoholic fatty liver disease or abdominal obesity.

**Variables**			**Model 1**			**Model 2**			**Model 3**		
	***n***	**(%)**	**Odds ratio**	**95% CI**	***p*-value**	**Odds ratio**	**95% CI**	***p* value**	**Odds ratio**	**95% CI**	***p*-value**
Total*(***n*** **=** **560)**											
NAFLD	400	71.4%	2.10	(1.69–2.62)	<0.001	3.00	(2.28–3.95)	<0.001	3.21	(2.18-4.73)	<0.001
AO	292	52.1%	2.72	(2.20–3.36)	<0.001	3.24	(2.41–4.36)	<0.001	2.92	(2.02-4.21)	<0.001
**Men (*****n*** **=** **480)**											
NAFLD	341	71.0%	2.69	(2.10–3.45)	<0.001	3.25	(2.33–4.55)	<0.001	3.26	(2.13-4.98)	<0.001
AO	234	48.8%	1.60	(1.25–2.06)	<0.001	2.43	(1.73–3.42)	<0.001	2.97	(1.91-4.62)	<0.001
**Women (*****n*** **=** **80)**											
NAFLD	59	73.8%	3.55	(2.08–6.07)	<0.001	3.37	(1.76–6.44)	<0.001	2.30	(1.07-4.94)	0.03
AO	58	72.5%	5.43	(3.19–9.25)	<0.001	6.41	(3.02–13.59)	<0.001	4.77	(2.01-11.34)	<0.001

### Risk of CACS > 0 in Subjects With Either NAFLD or AO

In the univariate analyses, the OR for CACS > 0 was much higher in the AO group than in the NAFLD group in both genders. However, in the adjusted model, the OR of CACS > 0 in subjects with NAFLD increased compared to subjects without NAFLD in men, but not in women (Table 3B). The OR for CACS > 0 was higher in subjects with AO compared with subjects without AO overall (Table 3B). In the multivariate analyses, after adjustment for age, DM, HTN, hyperlipidemia history, cigarette smoking, alcohol drinking, and exercise, the OR of the NAFLD and AO groups was attenuated, whereas the NAFLD group showed a relatively increased risk for CACS > 0 compared to that without NAFLD, and the OR was higher than that in men with AO as well [1.37; 95% confidence interval (CI) 1.03–1.83 vs. 1.35; 95% CI 1.02–1.79] (Table 3B). However, women with AO consistently had an increased OR for CACS > 0, while subjects with NAFLD showed a non-significantly increased OR for CACS > 0 (Table 3B). In the Supplementary Table 1B, after adjusting BMI variable, the results became no statistical difference. The VIF for each independent variable by checking multi-collinearity did not reach collinearity.

**Table 3B:** Odds ratio for coronary artery calcification (>0) in subjects with either non-alcoholic fatty liver disease or abdominal obesity.

**Variables**			**Model 1**			**Model 2**			**Model 3**		
	***n***	**(%)**	**Odds ratio**	**95% CI**	***p*-value**	**Odds ratio**	**95% CI**	***p* value**	**Odds ratio**	**95% CI**	***p*-value**
Total*(***n*** **=** **508)**											
NAFLD	339	66.7%	1.50	(1.21–1.87)	<0.001	1.43	(1.12–1.83)	0.004	1.39	(1.08–1.79)	0.01
AO	241	47.4%	1.94	(1.57–2.41)	<0.001	1.62	(1.27–2.05)	<0.001	1.46	(1.14–1.87)	0.003
**Men (*****n*** **=** **408)**											
NAFLD	280	68.6%	1.29	(1.00–1.67)	0.048	1.39	(1.05–1.85)	0.02	1.37	(1/03–1.83)	0.03
AO	179	43.8%	1.74	(1.36–2.24)	<0.001	1.51	(1.15–1.98)	0.003	1.35	(1.02–1.79)	0.04
**Women (*****n*** **=** **100)**											
NAFLD	59	59.0%	1.67	(1.07–2.61)	0.02	1.54	(0.94–2.54)	0.09	1.47	(0.87–2.47)	0.15
AO	62	62.0%	3.28	(2.08–5.17)	<0.001	1.97	(1.19–3.26)	0.01	1.87	(1.11–3.16)	0.02

Model 1, Unadjusted. Model 2, Adjusted for age. Model 3, Adjusted for age, HTN, DM, hyperlipidemia, smoking, alcohol drinking, and exercise. *Total group: model 2 and 3 added to adjust for sex.

### Risk for Intermediate to High CAD Risk (FRS ≥ 10%) in Groups by NAFLD and AO Status

Table 4A shows the association of FRS ≥ 10% with NAFLD and AO. In the univariate analysis, the OR for FRS ≥ 10% was highest in the group with both abnormalities, second highest in the group with AO only, and third highest in the group with NAFLD only. In the age-adjusted model, the OR for FRS ≥ 10% was the highest in the overall population and in men, but not in women, the AO-only group had a higher risk of FRS ≥ 10% than the NAFLD-only groups. In the multivariate analyses, the OR for FRS ≥ 10% was the highest in the group with both abnormalities (men: 5.86; 95% CI 3.37–10.20; women: 6.31; 95% CI 2.08–19.10). The group with NAFLD only and with both abnormalities showed a significantly increased OR for FRS ≥ 10% in men (2.21; 95% CI 1.32–10.20 and 5.86; 95% CI 3.37–10.20) (Table 4A). In contrast, in the NAFLD-only group, the risk of FRS ≥10% increased and remained statistically significant. In women, adjustment for factors increased the OR, and the OR for FRS ≥10% was significantly increased only in subjects with both NAFLD and AO (Table 4A). In the Supplementary Table 2A, after adjusting BMI variable, the results became no statistical difference. The VIF for each independent variable by checking multi-collinearity did not reach collinearity.

**Table 4A:** Odds ratio for Framingham risk score ≥ 10% (intermediate to high cardiovascular disease risk) in groups divided by non-alcoholic fatty liver disease and abdominal obesity status.

**Variables**	Total*			**Men**			**Women**		
	**Odds ratio**	**95% CI**	***p*-value**	**Odds ratio**	**95% CI**	***p*-value**	**Odds ratio**	**95% CI**	***p*-value**
**Model 1**									
NAFLD(−) AO(−)	1	-	-	1	-	-	1	-	-
NAFLD(−) AO(+)	2.55	(1.66–3.90)	<0.001	2.67	(1.53 −4.64)	<0.001	7.68	(2.99 −19.74)	<0.001
NAFLD(+) AO(−)	1.67	(1.26 −2.20)	<0.001	1.21	(0.89–1.65)	0.22	3.80	(1.54–9.40)	0.004
NAFLD(+) AO(+)	3.81	(2.90–5.00)	<0.001	3.05	(2.24–4.17)	<0.001	11.31	(5.12–24.96)	<0.001
**Model 2**									
NAFLD(−) AO(−)	1	-	-	1	-	-	1	-	-
NAFLD(−) AO(+)	2.08	(1.13–3.83)	0.02	2.65	(1.24–5.68)	0.01	2.39	(0.71–8.09)	0.16
NAFLD(+) AO(−)	2.03	(1.39–2.96)	<0.001	1.77	(1.18–2.67)	0.01	5.51	(1.78–17.04)	0.003
NAFLD(+) AO(+)	5.64	(3.84–8.29)	<0.001	4.92	(3.20–7.55)	<0.001	11.80	(4.34–32.05)	<0.001
**Model 3**									
NAFLD(−) AO(−)	1	-	-	1	-	-	1	-	-
NAFLD(−) AO(+)	1.50	(0.67–3.34)	0.32	2.46	(0.86–6.98)	0.09	1.06	(0.25–4.60)	0.94
NAFLD(+) AO(−)	2.20	(1.38–3.51)	0.001	2.21	(1.32–3.69)	0.002	2.94	(0.83–10.43)	0.09
NAFLD(+) AO(+)	5.69	(3.51–9.23)	<0.001	5.86	(3.37–10.20)	<0.001	6.31	(2.08–19.10)	0.001

### Risk of CACS > 0 in Groups by NAFLD and AO Status

Table 4B shows the association of CACS > 0 with NAFLD and AO. In the univariate analyses, the OR of CACS > 0 was highest in the pure AO group, second highest in the group with both abnormalities, and third highest in the pure NAFLD group. In the age-adjusted model, the OR for CACS > 0 was highest in the total population and in women, In contrast, among men, the risk of CACS > 0 was higher in the pure AO group than in the pure NAFLD group. In multivariate analysis, the OR of CACS > 0 was the highest among the groups with both abnormalities (men: 1.61; 95% CI 1.13–2.30; women: 2.17; 95% CI 1.13–4.16). The groups with NAFLD only and with both abnormalities showed a significantly increased OR for CACS >0 in men (1.41; 95% CI 1.05–1.99 and 1.61; 95% CI 1.13–2.30) (Table 4B). Although the risk of CACS > 0 was increased in the NAFLD-only group, this was attenuated in the AO-only group and remained statistically significant. In women, adjustment for factors attenuated the OR, with significantly increased OR for CACS > 0 only in subjects with both NAFLD and AO (Table 4B). In the Supplementary Table 2B, after adjusting BMI variable, the results became no statistical difference. The VIF for each independent variable by checking multi-collinearity did not reach collinearity.

**Table 4B:** Odds ratio for coronary artery calcification in groups divided by nonalcoholic fatty liver disease and abdominal obesity status.

**Variables**	Total^*^			**Men**			**Women**		
	**Odds ratio**	**95% CI**	***p*-value**	**Odds ratio**	**95% CI**	***p*-value**	**Odds ratio**	**95% CI**	***p*-value**
**Model 1**									
NAFLD(−) AO(−)	1	-	-	1	-	-	1	-	-
NAFLD(−) AO(+)	2.63	(1.73–4.01)	<0.001	2.38	(1.37–4.13)	0.002	4.47	(2.19–9.14)	<0.001
NAFLD(+) AO(−)	1.45	(1.10–1.92)	0.01	1.22	(0.89–1.67)	0.21	1.51	(0.75–3.01)	0.25
NAFLD(+) AO(+)	2.28	(1.74–2.99)	0.00	1.87	(1.37–2.57)	<0.001	3.58	(2.02–6.34)	<0.001
**Model 2**									
NAFLD(−) AO(−)	1	-	-	1	-	-	1	-	-
NAFLD(−) AO(+)	1.71	(1.07–2.73)	0.02	1.96	(1.06–3.63)	0.03	2.04	(0.91–4.60)	0.08
NAFLD(+) AO(−)	1.61	(1.20–2.18)	0.002	1.38	(0.98–1.94)	0.06	1.40	(0.66–2.95)	0.38
NAFLD(+) AO(+)	1.97	(1.47–2.64)	<0.001	1.77	(1.25–2.50)	0.001	2.31	(1.24–4.30)	0.01
**Model 3**									
NAFLD(−) AO(−)	1	-	-	1	-	-	1	-	-
NAFLD(−) AO(+)	1.55	(0.96–2.50)	0.07	1.75	(0.93–2.39)	0.08	1.97	(0.85–4.57)	0.11
NAFLD(+) AO(−)	1.59	(1.17–2.15)	0.003	1.41	(1.00–1.99)	0.05	1.36	(0.63–2.92)	0.43
NAFLD(+) AO(+)	1.75	(1.29–2.37)	<0.001	1.61	(1.13–2.30)	0.01	2.17	(1.13–4.16)	0.02

*Model 1, Unadjusted. Model 2, Adjusted for age. Model 3, Adjusted for age, HTN, DM, hyperlipidemia, smoking, alcohol drinking, and exercise*.

* *Total group: model 2 and 3 added to adjust for sex. Abbreviations as list in Table 1*.

## Discussion

Similar to previous studies (29, 31, 37, 38), our cross-sectional study was performed in a health-screening cohort and aimed to determine the relationship between prevalent NAFLD/AO and subclinical atherosclerosis/CAD risk. However, unique to our data was the finding that men with NAFLD have a significantly increased risk of CACS > 0 and FRS ≥ 10% compared to those with AO, whereas women with AO had a significantly increased risk for CACS > 0 and FRS ≥ 10%. In the Bonferroni *post-hoc* analysis, the mean ln(CACS + 1) or age/sex-adjusted mean ln(CACS + 1) was highest in subjects with AO-only group and lowest in subjects without any abnormality. The overall population with AO and NAFLD was significantly associated with CACS > 0 and FRS ≥ 10%. In summary, our results suggest that NAFLD is more closely associated with CACS > 0 and FRS ≥ 10% than AO in men, whereas AO is more closely associated with CACS > 0 and FRS ≥ 10% than NAFLD in women.

NAFLD is defined as the accumulation of more than 5% fat in hepatocytes in individuals whose alcohol intake is lower than 20 g/d (20–23). Although NAFLD is known as a hepatic manifestation of IR or MetS, several clinical studies have observed an association of NAFLD with CAC, independent of risk factors for cardiovascular disease such as IR, metabolic factors, and visceral adiposity tissue (37, 39–42). In support of the present findings in men, NAFLD, measured by magnetic resonance spectroscopy, was superior to visceral obesity, measured by magnetic resonance tomography, in determining increased carotid intima-media thickness, an estimate of early atherosclerosis, in subjects with prediabetes (43). In addition, studies have pointed out that genetically-driven NAFLD-related genes such as PNPLA3 and TM6SF2 genes are associated with increased liver fat content and progression to NASH and cirrhosis. However, these alleles are also unexpectedly associated with apparent protection from cardiovascular disease. Early detection of gene mutations will help the treatment of hepatic and extrahepatic diseases in different directions (44).

A previous study by Lee et al. (29) showed that AO measured via WHR was highly associated with CAC across the body in middle-aged Korean men. Low HC (decreased gluteo-femoral fat mass) is not only associated with NAFLD and AO, but is now also established as an important and independent risk factor of cardiometabolic diseases (17). Joo et al. (18) mentioned CT-measured WC using a fully automated body segmentation algorithm was closely correlated with manually-measured WC. Seimon et al. (19) found that WC was measured at the midpoint between the lowest rib and the iliac crest (WC_mid_) and the narrowest point of the torso (WC_narrow_) were gold-standard measurements which provided accurate estimations of visceral adipose tissue (VAT), as determined by MRI (Correlation *r* = 0.581and 0.563; *p* < 0.001 for all), especially for postmenopausal women with obesity. Our study also took the position of WC_mid_ for measurement, which has a correct correlation with VAT. Our results showed that AO measured via WC was strongly correlated with CAC in both genders. WC is an easier, convenience, and correct measurement of AO than HC or WHR, and based on our data, it can be considered an independent risk factor for CAC (16, 18, 19, 45).

Because FRS is easily calculated in the office setting, routine calculation of the FRS in NAFLD patients may be beneficial in identifying those NAFLD patients at highest risk of CHD outcomes (31). Treeprasertsuk et al. (31) suggested that a cut-point of 11 for women and 6 for men is the most sensitive cut-point for predicting CHD events. In this previous study, in these patients with NAFLD, the estimated 10-year CHD risk derived from the FRS (12.6% in men and 9.6% in women), respectively. For men with NAFLD, FRS is more sensitive than women, and patients with scores at or above these values may necessitate a more aggressive cardiac follow-up (31). In our study, we found a similar significant correlation with gender, and there was proportionally higher risk of CACS > 0 and FRS ≥ 10% among men with NAFLD than among women.

In this study, men and women with high WC (i.e., >90 and 80 cm, respectively) and without NAFLD were generally older and had higher blood pressure and fasting plasma glucose than subjects with NAFLD and without AO. Other studies have closely associated WC to hypertension and IR, which are well-known causes for CAC (46–48); WC was mentioned by Cassidy et al. (49) to be associated with the progression of subclinical coronary atherosclerosis. Our study results suggest that CACS was highest in men and women with AO only. After adjusting for known CAD risk factors such as age, chronic diseases, and lifestyle habits, patients with both NAFLD and AO had a synergistically increased risk for subclinical atherosclerosis and CAD risk. Moreover, after making the same adjustments, men with NAFLD only retained a significantly higher risk for CACS > 0 and FRS ≥10% than those with AO only. In contrast, women with AO only had a significantly higher risk for CACS > 0 and FRS ≥ 10% than those with NAFLD only. However, the higher risk for CAC and CAD risk in men with NAFLD only could possibly be attributed to higher prevalence of NAFLD in men (50). In a systematic review and meta-analysis, Balakrishnan et al. (50) found that women had a lower risk of NAFLD compared to men. From previous studies, it is understood that gender is one of the main determinants of body fat distribution (51, 52). Previous data has shown that visceral fat increases are greater in women than in men (51), which could be the one of the reasons for the higher risk for CAC and CAD risk in women with AO only. Our findings were consistent with those of a study by VanWagner et al. (38) performed in 2,424 participants, which suggested the importance of AO in the association of CAC and NAFLD Our results also support those of Lee et al. (29) which found that NAFLD was highly associated with CAC across the body in men.

Fat accumulation in hepatocytes and in the abdominal area are more closely associated with visceral adipose tissue (VAT) (45, 53). Increased VAT causes increased secretion of proinflammatory cytokines and adipokines, thus releasing free fatty acids into the body circulation system, which plays a role in the pathogenesis of NAFLD and AO (54, 55). Therefore, visceral fat accumulation might be a mediator linking NAFLD and AO to CAD (45, 54). Previous data have shown that visceral fat increases over 200% in men and 400% in women between the 3rd and 7th decades, owing to a shift from peripheral to central fat patterning. These studies have attempted to identify hormones that may be responsible for this shift (51, 52). These hormone or inflammation factors might play a role in the gender-based differences we found in our study.

Previous researchers seldom discussed the impact of BMI and excluded it in the multivariate analysis (29). The NAFLD/AO effect became non-significant after adjusting BMI in this study. This finding appeared not to be the impact of collinearity. We inferred BMI might confound the NAFLD/AO effect because BMI, AO, and NAFLD are all related to the inflammation of the body (29, 54–62). However, note a significant association between FRS and the NAFLD/AO (Supplementary Table) after adjusting BMI. We encouraged more future research to present more evidence to support this argument.

Previously, AO was consistently associated with NAFLD in 60–95% of cases, and AO assessed by WHR or WC was strongly associated with the prevalence of NAFLD (63, 64). AO was highly correlated with major cardiometabolic risk factors and established CVD, DM, or FRS ≧ 10%, suggesting it may be an easily measured surrogate for people at increased risk of future cardiovascular clinical events who may benefit from further assessment and intervention, especially for women (49). A case-control study recruiting 102 patients aged ≧60 years in Egypt showed that WC is a strong risk factor for hypertension and intermediate to high FRS score in Egyptian elderly women and not in men (65). Our study is consistent with these previous findings; In the absence of evidence of NAFLD, high WC may be an independent risk factor for CAD in women.

The strength of this study was to provide two methods to assess CAD risk, including CACS and FRS. The findings showed that NAFLD is more closely associated with CACS > 0 and FRS ≥ 10% than AO in men, whereas AO is more closely associated with CACS > 0 and FRS ≥ 10% than NAFLD in women. NAFLD and AO could be considered independent determinants of CACS by gender.

## Limitations

Our study has some potential limitations. First, our results might have varied according to the definition of WC (modified NCEP-ATP III for Taiwanese) (35, 36). It is unclear whether these results apply to races other than Taiwanese and to other East Asians. Second, a higher prevalence rate was found in Taiwan (11.5–52%) (23). It was as high as 80% in participants who were overweight or obese (66). In this study, the mean BMI was overweight, which resulted in higher prevalence of NAFLD (60.1%).This could have affected the relationship we found between NAFLD and CACS/FRS. Third, there were more men than women enrolled in the study, and the age of women were older than age of me in four groups, which may also cause differences in results [In Table 2, age-adjusted mean Ln (CACS+1) values differ from age- and sex-adjusted mean Ln (CACS+1) values)]. Ultimately, large-scale, prospective trials with a balanced proportion of men and women are needed to affirm our findings. Fourth, we also need to acknowledge the lack of assessment of proinflammatory cytokines, adipokines, or hormones (such as hs-CRP, IL-1, IL-6, or estrogen) (67), gene mutation (44), and hip circumference (17) in the current study. We were unable to further strengthen the role of these factors s in mediating atherosclerotic pathophysiology underlying excessive visceral adiposity. Fifth, determining alcohol intake history by self-questioning alone may introduce bias into the study. Sixth, US detects only moderate/severe hepatic steatosis. We use the double-blind progress of the operating physician to confirm the examination results, but in the future, we still need to use CT or MRI for liver fat confirmation (18, 19, 43). Finally, this study was cross-sectional design that did not allowto infer causality from the associations described. These subjects were from the Physical Examination Center to participate in this cardiovascular health survey, not from the clinical outpatient departments, which can reduce the selection error caused by existing diseases. However, the sample from a single tertiary medical center might still be due to differences in health habits and health concepts that caused Neyman bias. These limitations may be the reason why the ORs in Table 3 seems to be exaggerated in this study.

## Conclusion

In this health-screening population, Men with NAFLD only had a significantly higher risk of CAC and CAD than men with AO only, whereas this was higher in women with AO only than in women with NAFLD only after adjusting for age, chronic diseases, and lifestyle habits. Limited information was known about the exact gender-specific roles of NAFLD and AO as risk factors for CAD in previous studies. With the data from our study, we hope to determine the underlying pathogenesis related to the association of NAFLD and AO with CAD, and to establish effective lifestyle and drug treatment to prevent and reduce the occurrence of these diseases, ultimately reducing the risk of CAD in both men and women.

## Data Availability Statement

The raw data supporting the conclusions of this article will be made available by the authors, without undue reservation.

## Ethics Statement

The study protocol was evaluated and approved by the Human Research Ethics Committee of Mackay Memorial Hospital (project research number 18MMHIS137, 15 Oct 2018) and are in accordance with the Helsinki Declaration of 1975. Written informed consent for participation was not required for this study in accordance with the national legislation and the institutional requirements.

## Author Contributions

M-TT conceived and designed this study, performed cyroablation procedures, analyzed the data, contributed to data collection, and wrote the manuscript. J-YC carried subject's recruitment and data interpretation and performed cryoablation procedures. All authors contributed to the article and approved the submitted version.

## Conflict of Interest

The authors declare that the research was conducted in the absence of any commercial or financial relationships that could be construed as a potential conflict of interest.

## Publisher's Note

All claims expressed in this article are solely those of the authors and do not necessarily represent those of their affiliated organizations, or those of the publisher, the editors and the reviewers. Any product that may be evaluated in this article, or claim that may be made by its manufacturer, is not guaranteed or endorsed by the publisher.
