# Supplementary material for: Gender-Based Association of Coronary Artery Calcification and Framingham Risk Score With Non-alcoholic Fatty Liver Disease and Abdominal Obesity in Taiwanese Adults, a Cross-Sectional Study
Source: Front Cardiovasc Med. 2022 Mar 3;9:803967. doi: 10.3389/fcvm.2022.803967 (PMC8928543; doi:10.3389/fcvm.2022.803967)
Supplement: Supplementary file 1 [file Table_1.docx]

**Supplement table 1**

1. Odds ratio for Framingham risk score ≥ 10% in subjects with either nonalcoholic fatty liver disease or abdominal obesity

| **Variables** | |  |  |  | **Model 1** | | |  | **Model 2** | | |  | **Model 3** | | |  | **Model 4** | | |  |
| --- | --- | --- | --- | --- | --- | --- | --- | --- | --- | --- | --- | --- | --- | --- | --- | --- | --- | --- | --- | --- |
|  |  |  | **n** | **(%)** | **Odds ratio** | **95% CI** | **p value** |  | **Odds ratio** | **95% CI** | **p value** |  | **Odds ratio** | **95% CI** | **p value** |  | **Odds ratio** | **95% CI** | **p value** | **VIF** |
| **Total*** (n=560) | | | |  |  |  |  |  |  |  |  |  |  |  |  |  |  |  |  |  |
|  | NAFLD |  | 400 | 71.4% | 2.10 | (1.69-2.62) | <0.001 |  | 3.00 | (2.28-3.95) | <0.001 |  | 3.21 | (2.18-4.73) | <0.001 |  | 2.30 | (1.52-3.47) | <0.001 | 1.24 |
|  | AO |  | 292 | 52.1% | 2.72 | (2.20-3.36) | <0.001 |  | 3.24 | (2.41-4.36) | <0.001 |  | 2.92 | (2.02-4.21) | <0.001 |  | 1.62 | (1.01-2.61) | 0.045 | 1.90 |
| **Men** (n=480) | | | |  |  |  |  |  |  |  |  |  |  |  |  |  |  |  |  |  |
|  | NAFLD |  | 341 | 71.0% | 2.69 | (2.10-3.45) | <0.001 |  | 3.25 | (2.33-4.55) | <0.001 |  | 3.26 | (2.13-4.98) | <0.001 |  | 2.05 | (1.28-3.29) | 0.003 | 1.17 |
|  | AO |  | 234 | 48.8% | 1.60 | (1.25-2.06) | <0.001 |  | 2.43 | (1.73-3.42) | <0.001 |  | 2.97 | (1.91-4.62) | <0.001 |  | 1.77 | (1.02-3.07) | 0.04 | 1.91 |
| **Women** (n=80) | | | |  |  |  |  |  |  |  |  |  |  |  |  |  |  |  |  |  |
|  | NAFLD |  | 59 | 73.8% | 3.55 | (2.08-6.07) | <0.001 |  | 3.37 | (1.76-6.44) | <0.001 |  | 2.30 | (1.07-4.94) | 0.03 |  | 1.27 | (0.49-3.32) | 0.62 | 1.91 |
|  | AO |  | 58 | 72.5% | 5.43 | (3.19-9.25) | <0.001 |  | 6.41 | (3.02-13.59) | <0.001 |  | 4.77 | (2.01-11.34) | <0.001 |  | 3.71 | (1.47-9.36) | 0.01 | 1.36 |

1. Odds ratio for coronary artery calcification (> 0) in subjects with either nonalcoholic fatty liver disease or abdominal obesity

| **Variables** | |  |  |  | **Model 1** | | |  | **Model 2** | | |  | **Model 3** | | |  | **Model 4** | | |  |
| --- | --- | --- | --- | --- | --- | --- | --- | --- | --- | --- | --- | --- | --- | --- | --- | --- | --- | --- | --- | --- |
|  |  |  | **n** | **(%)** | **Odds ratio** | **95% CI** | **p value** |  | **Odds ratio** | **95% CI** | **p value** |  | **Odds ratio** | **95% CI** | **p value** |  | **Odds ratio** | **95% CI** | **p value** | **VIF** |
| **Total*** (n=508) | |  |  |  |  |  |  |  |  |  |  |  |  |  |  |  |  |  |  |  |
|  | NAFLD |  | 339 | 66.7% | 1.50 | (1.21-1.87) | <0.001 |  | 1.43 | (1.12-1.83) | 0.004 |  | 1.39 | (1.08-1.79) | 0.01 |  | 1.15 | (0.87-1.50) | 0.33 | 1.24 |
|  | AO |  | 241 | 47.4% | 1.94 | (1.57-2.41) | <0.001 |  | 1.62 | (1.27-2.05) | <0.001 |  | 1.46 | (1.14-1.87) | 0.003 |  | 1.02 | (0.73-1.42) | 0.92 | 1.90 |
| **Men** (n=408) | |  |  |  |  |  |  |  |  |  |  |  |  |  |  |  |  |  |  |  |
|  | NAFLD |  | 280 | 68.6% | 1.29 | (1.00-1.67) | 0.048 |  | 1.39 | (1.05-1.85) | 0.02 |  | 1.37 | (1/03-1.83) | 0.03 |  | 1.13 | (0.83-1.54) | 0.44 | 1,17 |
|  | AO |  | 179 | 43.8% | 1.74 | (1.36-2.24) | <0.001 |  | 1.51 | (1.15-1.98) | 0.003 |  | 1.35 | (1.02-1.79) | 0.04 |  | 0.85 | (0.58-1.25) | 0.41 | 1.90 |
| **Women** (n=100) | |  |  |  |  |  |  |  |  |  |  |  |  |  |  |  |  |  |  |  |
|  | NAFLD |  | 59 | 59.0% | 1.67 | (1.07-2.61) | 0.02 |  | 1.54 | (0.94-2.54) | 0.09 |  | 1.47 | (0.87-2.47) | 0.15 |  | 1.27 | (0.71-2.28) | 0.42 | 1.36 |
|  | AO |  | 62 | 62.0% | 3.28 | (2.08-5.17) | <0.001 |  | 1.97 | (1.19-3.26) | 0.01 |  | 1.87 | (1.11-3.16) | 0.02 |  | 1.79 | (0.91-3.51) | 0.09 | 1.91 |

Model 1: Unadjusted.

Model 2: Adjusted for age.

Model 3: Adjusted for age, HTN, DM, hyperlipidemia, smoking, alcohol drinking, and exercise.

Model 4: Adjusted for age, sex, HTN, DM, Hyperlipidemia, smoking, alcohol drinking, exercise and BMI.

* Total group: model 2 and 3 added to adjust for sex.

VIF: variance inflation factor

**Supplement table 2**

1. Odds ratio for Framingham risk score ≥ 10% (intermediate to high cardiovascular disease risk) in groups divided by nonalcoholic fatty liver disease and abdominal obesity status

| **Variables** | |  | **Total*** | | | | **Men** | | | | **Women** | | | |
| --- | --- | --- | --- | --- | --- | --- | --- | --- | --- | --- | --- | --- | --- | --- |
|  |  |  | **Odds ratio** | **95% CI** | **p value** | **VIF** | **Odds ratio** | **95% CI** | **p value** | **VIF** | **Odds ratio** | **95% CI** | **p value** | **VIF** |
| Model 1 | |  |  |  |  |  |  |  |  |  |  |  |  |  |
|  | NAFLD(-) AO(-) |  | 1 | - | - |  | 1 | - | - |  | 1 | - | - |  |
|  | NAFLD(-) AO(+) |  | 2.55 | (1.66-3.90) | <0.001 |  | 2.67 | (1.53-4.64) | <0.001 |  | 7.68 | (2.99-19.74) | <0.001 |  |
|  | NAFLD(+) AO(-) |  | 1.67 | (1.26-2.20) | <0.001 |  | 1.21 | (0.89-1.65) | 0.22 |  | 3.80 | (1.54-9.40) | 0.004 |  |
|  | NAFLD(+) AO(+) |  | 3.81 | (2.90-5.00) | <0.001 |  | 3.05 | (2.24-4.17) | <0.001 |  | 11.31 | (5.12-24.96) | <0.001 |  |
| Model 2 | |  |  |  |  |  |  |  |  |  |  |  |  |  |
|  | NAFLD(-) AO(-) |  | 1 | - | - |  | 1 | - | - |  | 1 | - | - |  |
|  | NAFLD(-) AO(+) |  | 2.08 | (1.13-3.83) | 0.02 |  | 2.65 | (1.24-5.68) | 0.01 |  | 2.39 | (0.71-8.09) | 0.16 |  |
|  | NAFLD(+) AO(-) |  | 2.03 | (1.39-2.96) | <0.001 |  | 1.77 | (1.18-2.67) | 0.01 |  | 5.51 | (1.78-17.04) | 0.003 |  |
|  | NAFLD(+) AO(+) |  | 5.64 | (3.84-8.29) | <0.001 |  | 4.92 | (3.20-7.55) | <0.001 |  | 11.80 | (4.34-32.05) | <0.001 |  |
| Model 3 | |  |  |  |  |  |  |  |  |  |  |  |  |  |
|  | NAFLD(-) AO(-) |  | 1 | - | - |  | 1 | - | - |  | 1 | - | - |  |
|  | NAFLD(-) AO(+) |  | 1.50 | (0.67-3.34) | 0.32 |  | 2.46 | (0.86-6.98) | 0.09 |  | 1.06 | (0.25-4.60) | 0.94 |  |
|  | NAFLD(+) AO(-) |  | 2.20 | (1.38-3.51) | 0.001 |  | 2.21 | (1.32-3.69) | 0.002 |  | 2.94 | (0.83-10.43) | 0.09 |  |
|  | NAFLD(+) AO(+) |  | 5.69 | (3.51-9.23) | <0.001 |  | 5.86 | (3.37-10.20) | <0.001 |  | 6.31 | (2.08-19.10) | 0.001 |  |
| Model 4 | |  |  |  |  |  |  |  |  |  |  |  |  |  |
|  | NAFLD(-) AO(-) |  | 1 | - | - |  | 1 | - | - |  | 1 | - | - |  |
|  | NAFLD(-) AO(+) |  | 0.98 | (0.41-2.32) | 0.96 | 1.36 | 1.48 | (0.49-4.46) | 0.49 | 1.20 | 0.76 | (0.15-3.78) | 0.74 | 1.45 |
|  | NAFLD(+) AO(-) |  | 1.94 | (1.20-3.12) | 0.01 | 1.45 | 1.92 | (1.14-3.26) | 0.02 | 2.04 | 2.55 | (0.70-9.27) | 0.16 | 1.31 |
|  | NAFLD(+) AO(+) |  | 3.28 | (1.78-6.05) | <0.001 | 2.59 | 3.22 | (1.60-6.50) | 0.001 | 1.38 | 4.00 | (1.02-15.75) | 0.05 | 2.59 |

1. Odds ratio for coronary artery calcification in groups divided by nonalcoholic fatty liver disease and abdominal obesity status

| **Variables** | | | **Total*** | | | | **Men** | | | | **Women** | | | |
| --- | --- | --- | --- | --- | --- | --- | --- | --- | --- | --- | --- | --- | --- | --- |
|  |  |  | **Odds ratio** | **95% CI** | **p value** | **VIF** | **Odds ratio** | **95% CI** | **p value** | **VIF** | **Odds ratio** | **95% CI** | **p value** | **VIF** |
| Model 1 | |  |  |  |  |  |  |  |  |  |  |  |  |  |
|  | NAFLD(-) AO(-) |  | 1 | - | - |  | 1 | - | - |  | 1 | - | - |  |
|  | NAFLD(-) AO(+) |  | 2.63 | (1.73-4.01) | <0.001 |  | 2.38 | (1.37-4.13) | 0.002 |  | 4.47 | (2.19-9.14) | <0.001 |  |
|  | NAFLD(+) AO(-) |  | 1.45 | (1.10-1.92) | 0.01 |  | 1.22 | (0.89-1.67) | 0.21 |  | 1.51 | (0.75-3.01) | 0.25 |  |
|  | NAFLD(+) AO(+) |  | 2.28 | (1.74-2.99) | 0.00 |  | 1.87 | (1.37-2.57) | <0.001 |  | 3.58 | (2.02-6.34) | <0.001 |  |
| Model 2 | |  |  |  |  |  |  |  |  |  |  |  |  |  |
|  | NAFLD(-) AO(-) |  | 1 | - | - |  | 1 | - | - |  | 1 | - | - |  |
|  | NAFLD(-) AO(+) |  | 1.71 | (1.07-2.73) | 0.02 |  | 1.96 | (1.06-3.63) | 0.03 |  | 2.04 | (0.91-4.60) | 0.08 |  |
|  | NAFLD(+) AO(-) |  | 1.61 | (1.20-2.18) | 0.002 |  | 1.38 | (0.98-1.94) | 0.06 |  | 1.40 | (0.66-2.95) | 0.38 |  |
|  | NAFLD(+) AO(+) |  | 1.97 | (1.47-2.64) | <0.001 |  | 1.77 | (1.25-2.50) | 0.001 |  | 2.31 | (1.24-4.30) | 0.01 |  |
| Model 3 | |  |  |  |  |  |  |  |  |  |  |  |  |  |
|  | NAFLD(-) AO(-) |  | 1 | - | - |  | 1 | - | - |  | 1 | - | - |  |
|  | NAFLD(-) AO(+) |  | 1.55 | (0.96-2.50) | 0.07 |  | 1.75 | (0.93-2.39) | 0.08 |  | 1.97 | (0.85-4.57) | 0.11 |  |
|  | NAFLD(+) AO(-) |  | 1.59 | (1.17-2.15) | 0.003 |  | 1.41 | (1.00-1.99) | 0.05 |  | 1.36 | (0.63-2.92) | 0.43 |  |
|  | NAFLD(+) AO(+) |  | 1.75 | (1.29-2.37) | <0.001 |  | 1.61 | (1.13-2.30) | 0.01 |  | 2.17 | (1.13-4.16) | 0.02 |  |
| Model 4 | |  |  |  |  |  |  |  |  |  |  |  |  |  |
|  | NAFLD(-) AO(-) |  | 1 | - | - |  | 1 | - | - |  | 1 | - | - |  |
|  | NAFLD(-) AO(+) |  | 1.16 | (0.77-1.75) | 0.48 | 2.59 | 1.26 | (0.89-1.80) | 0.69 | 1.20 | 1.99 | (0.79-4.97) | 0.14 | 1.45 |
|  | NAFLD(+) AO(-) |  | 1.28 | (0.93-1.77) | 0.13 | 1.45 | 0.97 | (0.61-1.55) | 0.20 | 2.03 | 1.36 | (0.62-2.99) | 0.44 | 1.31 |
|  | NAFLD(+) AO(+) |  | 1.34 | (0.79-2.28) | 0.29 | 1.36 | 1.15 | (0.58-2.27) | 0.90 | 1.38 | 2.19 | (0.92-5.21) | 0.08 | 2.60 |

Model 1: Unadjusted.

Model 2: Adjusted for age.

Model 3: Adjusted for age, HTN, DM, hyperlipidemia, smoking, alcohol drinking, and exercise.

Model 4: Adjusted for age, HTN, DM, hyperlipidemia, smoking, alcohol drinking, exercise, and BMI.

* Total group: model 2 and 3 added to adjust for sex.

Abbreviations as list in table 1

VIF: variance inflation factor
